# Effect of Pore Size Distribution on Energy Storage of Nanoporous Carbon Materials in Neat and Dilute Ionic Liquid Electrolytes

**DOI:** 10.3390/molecules28207191

**Published:** 2023-10-20

**Authors:** Maike Käärik, Mati Arulepp, Anti Perkson, Jaan Leis

**Affiliations:** 1 Institute of Chemistry, University of Tartu, Ravila 14a, 50411 Tartu, Estonia; 2 Skeleton Technologies, Sepise 7, 11415 Tallinn, Estonia

**Keywords:** nanoporous carbon, carbide-derived carbon, pore size distribution, electrical double-layer capacitor, ionic liquid electrolytes

## Abstract

This study investigates three carbide-derived carbon (CDC) materials (TiC, NbC, and Mo_2_C) characterized by uni-, bi-, and tri-modal pore sizes, respectively, for energy storage in both neat and acetonitrile-diluted 1-ethyl-3-methylimidazolium bis(trifluoromethylsulfonyl)imide. A distribution of micro- and mesopores was studied through low-temperature N_2_ and CO_2_ adsorption. To elucidate the relationships between porosity and the electrochemical properties of carbon materials, cyclic voltammetry, galvanostatic cycling, and electrochemical impedance spectroscopy measurements were conducted using three-electrode test cells. The ultramicroporous TiC-derived carbon is characterized by a high packing density of 0.85 g cm^−3^, resulting in superior cathodic and anodic capacitances for both neat ionic liquid (IL) and a 1.9 M IL/acetonitrile electrolyte (93.6 and 75.8 F cm^−3^, respectively, in the dilute IL). However, the bi-modal pore-sized microporous NbC-derived carbon, with slightly lower cathodic and anodic capacitances (i.e., 85.0 and 73.7 F cm^−3^ in the dilute IL, respectively), has a lower pore resistance, making it more suitable for real-world applications. A symmetric two-electrode capacitor incorporating microporous CDC-NbC electrodes revealed an acceptable cycle life. After 10,000 cycles, the cell retained approximately 75% of its original capacitance, while the equivalent series resistance (ESR) only increased by 13%.

## 1. Introduction

The increasing global interest in high-performance energy storage devices has spurred the exploration of various electrochemical storage solutions, including electrostatic, redox, and hybrid storage systems. Among these, electrostatic energy storage devices, commonly known as ultracapacitors (or supercapacitors), stand out as the most environmentally friendly option with the longest cycle life. These devices comprise large-surface carbon electrodes that can endure thousands of charge and discharge cycles. Their exceptionally low internal resistance contributes to their outstanding power characteristics, giving them a significant advantage over redox storage devices like secondary batteries. However, a major challenge faced by ultracapacitor developers is the relatively low energy density, which remains over 20 times lower than that of advanced lithium batteries.

The energy storage mechanism in ultracapacitors involves ion electrosorption on the surface of porous carbon electrodes. In general, a fundamental principle dictates that the larger the surface area accessible to the electrolyte, the greater the device’s capacity [1]. Nanoporous carbon stands out as the most appealing electrode material, primarily due to its high surface-area-to-volume ratio. The impact of pore sizes on the capacitance of carbon materials has garnered significant attention. Research has established that a higher capacitance results from optimal matching between electrolyte ions and carbon pore sizes [1,2,3,4]. Carbide-derived carbon (CDC), a well-recognized representative of nanoporous carbons, has consistently demonstrated its suitability for ultracapacitor applications [5,6]. The structure and porosity of CDC can be meticulously controlled by adjusting the structure and composition of the precursor carbide and the synthesis conditions [2]. Moreover, the textural properties of CDC can be finely adjusted during post-treatment using either chemical methods [7,8,9] or physical activation techniques [10,11,12,13].

In accordance with the general definition of capacitance, a larger specific surface area corresponds to a higher capacitance. However, it is crucial to consider that this relationship is typically non-linear for materials with larger specific surface areas [14,15]. Therefore, it is essential to account for the pore sizes of the material as well [2,3,16,17]. In recent years, several studies [2,3,18,19] have introduced combined models for micro- and mesoporous carbon materials to illustrate the relationship between capacitance and pore size. These studies have demonstrated that both gravimetric [2,18] and volumetric [2,3] capacitance can be effectively simulated using data obtained from CO_2_ and N_2_ adsorption experiments.

The primary requirements for ultracapacitor electrolytes include high ionic conductivity, chemical and electrochemical stability, a wide operating temperature range, low resistance, and environmental compatibility [20]. Liquid electrolytes can be broadly categorized into non-aqueous, aqueous, and ionic liquid electrolytes [20,21].

Finding an electrolyte that fulfills all requirements can be quite challenging. Aqueous electrolytes offer high conductivity and capacitance but suffer from low energy density due to a narrow applicable voltage window (approximately 1 V). In contrast, organic (non-aqueous) electrolytes and ionic liquids (ILs) can operate at higher voltages but generally exhibit lower ionic conductivity [20,22]. One drawback of organic electrolytes is the larger size of their ions, which increases the specific resistance compared to water-based electrolytes and necessitates electrodes with larger pore sizes, thus reducing the electrode density [22]. Nevertheless, owing to their broader applicable voltage window (2.6–2.9 V), organic electrolytes, primarily based on quaternary ammonium salts in propylene carbonate (PC) or acetonitrile (ACN), currently dominate the field of commercial storage devices [1,22,23].

Ionic liquids, which consist of asymmetric organic cations and anions, offer several advantages over organic electrolytes. They exhibit high chemical, thermal, and electrochemical stability, are non-flammable, and permit the use of a higher electrochemical stability window (ESW), typically exceeding 3 V. In accordance with the characteristics commonly observed in organic electrolytes, it is noteworthy that the reduction in ionic size within ionic liquids (ILs) corresponds to decreased electrical resistance and an increase in specific capacitance. This principle has been explored in previous studies, such as those referenced in [24,25], in which the influence of cation size in IL electrolytes was investigated, and in [26], which delves into the electrochemical performance of various anions. Furthermore, an investigation was conducted employing three distinct ionic liquids (EMIm-TFSI, EMIm-BF_4_, and BMIm-PF_6_) in conjunction with porous carbon electrodes characterized by an average pore size of approximately 1.2 nm. This study revealed that smaller ions are more involved in the charging mechanism near the potential of zero charge (pzc), whereas at higher potential values, the mechanism shifts toward preferred counterion adsorption [27].

A notable drawback of IL-based electrolytes is their high viscosity, especially at lower temperatures [1,20,23,28,29]. In order to address the viscosity challenge, ionic liquids can be blended with low-viscosity organic solvents like PC or ACN [30]. Using a mixture of 1-ethyl-3-methylimidazolium bis(trifluoromethylsulfonyl)imide (EMIm-TFSI) and ACN, mesopores were shown to facilitate fast ion transport in carbon electrodes, achieving a specific capacitance of up to 130 F g^−1^ [31].

Largeot et al. investigated the relationship between carbon pore size and ion size while employing EMIm-TFSI as an electrolyte. In this context, EMIm^+^ and TFSI^−^ ions are roughly comparable in size (0.7 and 0.79 nm in their longest dimensions). Their study revealed that the maximum capacitance of 160 F g^−1^ (calculated from a galvanostatic experiment conducted at 5 mA cm^−2^ in a neat EMIm-TFSI electrolyte at 60 °C using a voltage window of 0–3 V) was attained with carbon possessing an average pore width of 0.72 nm. This dimension closely matches the size of the EMIm^+^ and TFSI^−^ ions, resulting in optimal ion adsorption efficiency [4]. A separate study [32], employing TiC-derived CDC as the electrode and a 2 M EMIm-TFSI/ACN mixture as the electrolyte in a three-electrode configuration, also concluded that the maximum capacitance is achieved when the pore size of the carbon closely aligns with the ion size. In smaller nanopores, ion adsorption, especially of larger TFSI anions, was diffusion-controlled due to hindered accessibility caused by size effects. A similar observation was reported regarding ion sizes and sub-nanopore matching for EMIm-TFSI and EMIm-BF_4_, where secondary carbon nanopores of 0.50 nm were exclusively accessible to small BF_4_^−^ anions (0.48 nm) but not to larger TFSI^−^ ions (0.79 nm) [33].

The interaction between ionic liquid structures and various carbon materials and their consequential effects have been the subject of prior investigations [34]. These studies emphasized that the selection of a carbon electrode should not solely be based on pore size and surface area; it should also consider the interaction between the surface chemistry of the electrode and the electrolyte. While materials with high specific surface areas, such as mesoporous and activated carbon, exhibited respectable specific capacitance and energy density, materials resembling graphene and carbon nanotubes with open surfaces demonstrated superior power density.

Härmas et al. have reported that ultramicroporous materials are unsuitable for high-power and high-energy-density supercapacitors due to the slow establishment of adsorption equilibrium in viscous ionic liquids [35]. However, when the viscosity of the ionic liquid is reduced by introducing an additional solvent like ACN, ultramicroporous materials become exceedingly attractive due to the increased density at the corresponding electrodes, resulting in enhanced volumetric energy and power density [36].

Previously, the interconnectedness of porosity parameters in carbon materials and their utility in quantitatively predicting EDLC across various organic electrolytes were demonstrated [2,3]. In the present study, a preliminary investigation of three CDC materials, originating from TiC, NbC, and Mo_2_C, characterized by unimodal, bimodal, and trimodal pore size distributions, respectively, is undertaken. This investigation encompasses energy storage within both undiluted and diluted ILs. The primary objective is to assess whether the energy storage properties of ultramicroporous carbon materials, akin to the majority of CDCs, are enhanced in IL environments due to the presence of larger transport pores within the carbon material structure.

## 2. Results and Discussion

The three carbon materials (CDCs) with variable pore size distributions used in this study were made by the chlorination of different metal carbides: TiC at a temperature of 500 °C [10], Mo_2_C at a temperature of 800 °C [37], and NbC at a temperature of 1000 °C [2]. The CDCs are distinguished as CDC-TiC, CDC-NbC, and CDC-Mo_2_C, respectively.

**Figure 1:**
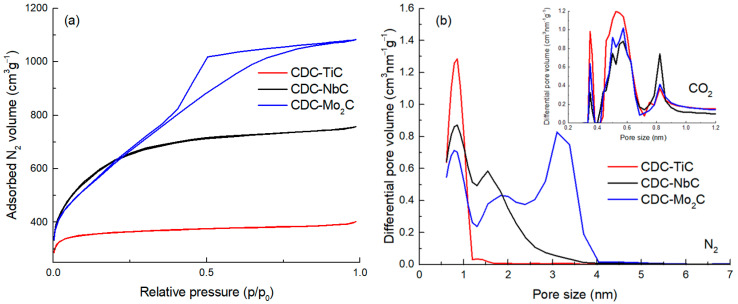
N_2_ adsorption isotherms (**a**) and the corresponding pore size distribution of CDC materials calculated from N_2_ and CO_2_ isotherms (**b**).

As previously shown [2,38], the structural order and porosity of CDCs are related to the structure of the precursor carbide and the temperature of chlorination; the higher the temperature, the higher the degree of graphitization and the larger the average pore size. The microstructure has also been studied in CDCs derived from TiC [10,39], NbC [39], and Mo_2_C [37,40]. In Figure 1a, one can observe that the N_2_ adsorption isotherm for CDC-TiC corresponds to Type I (a) by IUPAC [41], characteristic of microporous materials with a narrow pore size distribution. The CDC-NbC exhibits a Type I (b) isotherm, which shows a broader pore size distribution in the micropore region. The CDC synthesized from Mo_2_C (i.e., CDC-Mo_2_C) corresponds to Type IV (a), with a noticeable hysteresis loop indicating the presence of mesopores.

The pore size distributions (PSDs) were calculated from the N_2_ isotherms and are presented in Figure 1b. The PSD of all three CDCs contains a peak with pore sizes around 0.8 nm. CDC-NbC contains additional pores at 1.6 nm. CDC-Mo_2_C represents micromesoporous carbon, whose PSD contains, in addition to micropores at 0.8 and 1.9 nm, a considerable amount of mesopores at 3 nm.

The texture characteristics of the CDCs are collected in Table 1. Mo_2_C-CDC reveals the largest specific surface area as well as total porosity (2204 m^2^g^−1^ and 1.67 cm^3^g^−1^, respectively), whereas CDC-NbC has the most significant amount of micropores (0.90 cm^3^g^−1^). Among all three CDCs, CDC-TiC has a majority of pores smaller than 1 nm. Also, the PSD calculated from CO_2_ adsorption (inset on Figure 1b) confirms that the carbon made of TiC has the highest proportion of sub-nanometer-sized pores.

An electrochemical evaluation of the CDC electrodes was performed in a three-electrode test cell using the neat ionic liquid EMIm-TFSI as the electrolyte at 60 °C and a 1.9 M EMIm-TFSI/ACN mixture at room temperature. A cyclic voltammetry (CV) study was performed over a potential range of −1.4 V to +1.4 V vs. a carbon electrode.

**Figure 2:**
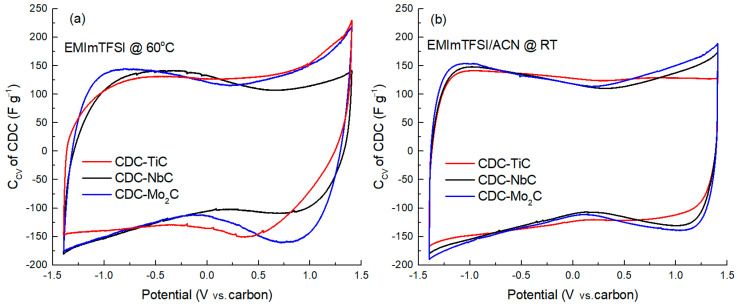
Cycling voltammetry curves of CDC materials in neat IL (**a**) and IL/ACN mixture (**b**), expressed as capacitance (Ccv) vs. potential at a scan rate of 1 mV s^−1^.

Figure 2 presents the cyclic voltammetry curves of the CDC materials measured at a voltage scan rate of 1 mV s^−1^ in a potential window of −1.4 V to +1.4 V. A visual examination of these graphs (Figure 2a,b) reveals a characteristic flat minimum in the central region of the curve, near 0 V, corresponding to the zero-charge potential of carbon. This is caused by the fact that, as the electrode potential increases, the number of electrosorbed ions on the surface increases too, which leads to an increase in the density of the electric charge on the electrode surface, resulting in an increase in the capacitance of the electric double layer. This minimum is located somewhat differently on various CDC materials, with the flatness of the minimum increasing as the carbon material becomes more microporous. For example, in the case of CDC-TiC in the EMIm-TFSI/ACN electrolyte, no zero-charge minimum is observed at all. The flatness of this minimum is influenced by the electrolyte viscosity, ion dimensions, and the micromesoporous nature of the carbon (cf. Table 1).

In addition to the minimum, the curves can be characterized by the current response upon potential reversal, specifically the “backswing.” For example, it is evident that after the potential reversal (after +1.4 V or −1.4 V) in the neat EMIm-TFSI (Figure 2a), the curves exhibit a flatter curvature compared to dilute electrolyte solutions (Figure 2b). This is again due to the significantly higher viscosity of the neat ionic liquid. Additionally, there is an observable dependence of the slope on the carbon material, where a smaller average pore size (1.23 nm) of CDC-NbC compared to 1.74 nm of CDC-Mo_2_C leads to a shallower slope. For instance, a distinctive change in the slope (after potential reversal at +1.4 V) is observed in cells with the neat IL electrolyte, where the slope increases in the sequence CDC-TiC, CDC-NbC, and CDC-Mo_2_C. However, in the cathodic (negative potential) region, CDC-TiC stands out due to its very high impedance at negative potentials, which is “amplified” by the high viscosity of the IL and its low conductivity. Therefore, a change in potential causes a sharp current drop to 0 and distorts the CV curve shape. In dilute electrolytes, the slope after potential reversal is steeper, primarily due to the lower electrolyte viscosity and higher conductivity mentioned earlier.

In the mixed IL/ACN electrolyte, when comparing the CV curves of CDC-NbC and CDC-Mo_2_C (Figure 2b), they are quite similar, with CDC-TiC, the most microporous, showing some differences. This may be attributed to the ion solvation effects and slightly larger ion dimensions in the EMIm-TFSI/ACN electrolyte compared to the neat IL. Furthermore, the lower anodic capacitance of the CDC-TiC material supports this hypothesis, influenced by the larger dimensions of TFSI^−^ anions compared to EMIm^+^ cations (0.6 × 0.9 nm vs. 0.5 × 0.8 nm [42]).

**Figure 3:**
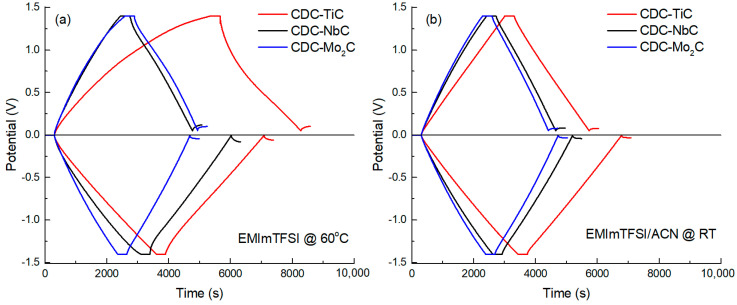
CC charge–discharge curves (I = 0.5 mA cm^−2^) of CDC materials in neat IL (**a**) and IL/ACN mixture (**b**).

To evaluate the EDL capacitance of CDCs at different current densities (0.5 to 5 mA cm^−2^), constant current (CC) charge–discharge measurements were conducted in the anodic and cathodic potential ranges. A 5 min holding at the operating potential was applied in order to achieve electrosorption equilibrium (i.e., maximum charging) at the given potential, consequently leading to better reproducibility of the capacitance values. Characteristic CC curves are shown in Figure 3, at a current density of 0.5 mA cm^−2^ and potential ranges of 0 to +1.4 V and 0 to −1.4 V. In Figure 3, CDC-TiC, the most microporous material, stands out somewhat at anodic potentials, manifesting as non-linear charge–discharge curves in the neat IL electrolyte. This behavior is due to the high viscosity of the neat IL (14.2 cP [43]) and the interaction between somewhat larger electrolyte anions and the sub-nanometer-sized pores. After diluting the IL with ACN, the viscosity of the electrolyte significantly decreases (by ~10-fold [43]), and the non-linearity of the charge–discharge curves of CDC-TiC disappears.

Table 2 provides the specific capacitance values of all three CDCs in this study, calculated using the constant current method and measured in a neat ionic liquid electrolyte at 60 °C. Comparing these values to the gravimetric capacitances, it can be observed that CDC-Mo_2_C, with the largest pore dimensions, has nearly equal cathodic and anodic capacitances of 140 vs. 146 F g^−1^. A similar level of 145 F g^−1^ is achieved by CDC-NbC in the cathodic direction. This uniform level is a result of these materials’ pore size and ion dimensions having an almost “ideal” fit in the IL. Moreover, even the high viscosity of the electrolyte does not impose limitations on achieving this capacitance level.

The lower capacitance of the most microporous material (CDC-TiC) is due to its lower specific surface area and significantly lower porosity. The anodic capacitance of CDC-TiC is lower because the pore dimensions of its carbon are very close to the size of the electrolyte anions, and not all micropores are accessible to the electrolyte ions. However, the limited access to the micropores is compensated by the very high carbon packing density in the case of CDC-TiC, which therefore reveals the highest volumetric capacitance values, ranging from 89 to 100 F cm^−3^ for the anodic and cathodic capacitance, respectively. In contrast, CDC-NbC, which has significantly higher overall porosity and surface area than CDC-TiC, exhibits lower density and, therefore, lower volumetric capacitance compared to CDC-TiC. As a rule, the larger the pore volume, the lower the material density (as more pores result in a decrease in material density). 

When comparing the capacitance values in the neat IL (Table 2) and the diluted electrolyte (Table 3), it is evident that ion solvation by ACN shifts the capacitance values in both directions, sometimes increasing and sometimes decreasing. For example, the cathodic capacitance is increased in CDC-TiC (in the dilute electrolyte compared to the neat IL), most probably due to a decrease in electrolyte viscosity, which improves ion diffusion in the micropores, while the dilution of IL has a negligible effect on the anodic capacitance, which is practically the same in both electrolytes (113 F g^−1^).

CDC-NbC behaves differently from TiC in terms of capacitance. In the dilute IL, CDC-NbC cathodic capacitance is lower (138 vs. 145 F g^−1^) compared to the solvent-free environment. However, in the case of anodic capacitance, the situation is reversed, with ~120 F g^−1^ vs. 110 F g^−1^ for the IL/ACN and neat IL, respectively. This shows that carbon materials with sufficiently large micropores are suitable for achieving high capacitance in ionic liquids, but in a solvent-diluted environment, the solvation of ions begins to limit the capacitance, which is particularly pronounced in NbC at the anodic potential.

When comparing the capacitances of CDC-Mo_2_C in two different electrolytes (see Table 2 and Table 3), it appears that its large pores are not limiting and are suitable for cathodic potential, but a capacitance decrease occurs at the anodic potential in the solvent-diluted environment (i.e., capacitance decreases from 146 F g^−1^ to 128 F g^−1^). However, CDC-Mo_2_C provides valuable information due to its very large pore dimensions when fitting with the solvent effects and pore dimensions in the electrolyte. Nevertheless, CDC-Mo_2_C has significantly lower volumetric capacitance (60–66 F cm^−3^) in comparison to the other materials studied, making it less practical for EDL devices due to its low energy density.

**Figure 4:**
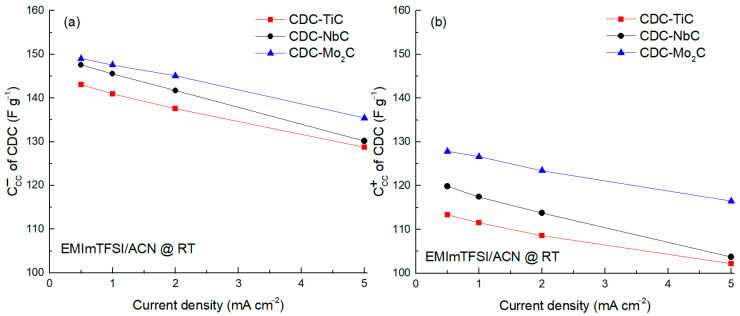
Specific capacitance (*C*_cc_) of carbon materials calculated at negative −1.6 V (**a**) and positive 1.4 V (**b**) potentials at different current densities.

The analysis of the CC capacitance vs. current density in the range 0.5–5.0 mA cm^−2^ was performed using ACN-diluted IL (cf. Figure 4). The findings from this study confirm that the cathodic capacitance, attributed to the electrosorption of EMIm^+^ ions, surpasses the anodic capacitance by approximately 20 F g^−1^. This phenomenon is particularly notable due to the larger dimensions of the TFSI^−^ anions. Furthermore, the influence of the ion size becomes evident in the substantial discrepancy in capacitance observed between the CDCs at the anode. This discrepancy suggests restricted accessibility to micropores when the dimensions of the ions closely approach those of the micropores themselves.

**Figure 5:**
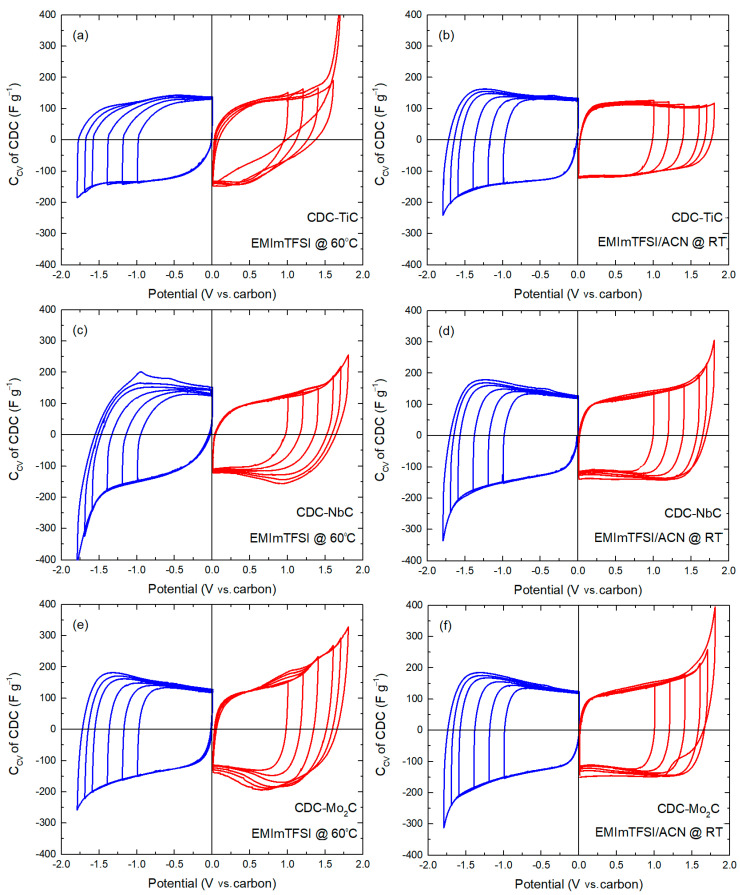
Cycling voltammetry curves (v = 1 mV s^−1^) for positively (red) and negatively (blue) charged electrodes expressed as gravimetric capacitance (*C*_CV_) for CDC-TiC (**a**,**b**), CDC-NbC (**c**,**d**), and CDC-Mo_2_C-CDC (**e**,**f**). Electrolyte and measurement temperature are shown on each graph.

Cyclic voltammetry was utilized to investigate the behavior of electrode materials at positive and negative electrode potentials. To ascertain electrochemical stability, measurements were conducted over a wide electrochemical window, i.e., +1.8 V to 0 V and −1.8 V to 0 V vs. a carbon reference electrode. The CV diagrams for the materials studied are presented in Figure 5.

Evaluating the electrochemical stability of materials is essential to determining the voltage limits. As these limits are not the same for different carbons, electrochemical window studies were conducted separately at positive and negative electrode potentials. In this process, the electrode potential was incrementally increased, starting from 1.0 V with a 200 mV step and gradually reaching 1.6 V, followed by smaller 100 mV increments up to 1.8 V. 

For the CDC-TiC material, its cathodic curves in the neat ionic liquid are relatively similar, and starting from −1.6 V, there is a gradual increase in current during charging (i.e., an increase in capacitance). Such an increase in current results in a flat peak in the reverse scan of the CV cycle in the −0.8 V to −0.6 V range, which slightly grows with an expanding CV potential window, indicating a partially reversible process.

In the positive potential region for the CDC-TiC material, the CV curves up to +1.4 V are relatively similar. With further potential increases beyond +1.6 V, the polarization current increases rapidly, indicating electrolyte decomposition and potential partial surface blocking by system degradation products. This process becomes especially evident in the curve of +1.7 V. Therefore, the shape of the discharge at +1.7 V polarizations is significantly different from lower potentials.

In the case of the EMIm-TSI/ACN electrolyte solution, the anodic end of the CV curve of CDC-TiC is very stable and almost rectangular in shape. The complete absence of peaks in the presence of ACN (Figure 5b) suggests that adding a solvent significantly improves the stability of the carbon/electrolyte interface. In the cathodic CV polarization curves in the dilute IL electrolyte, there is a similar trend as seen in the full-scan curves; achieving capacitance during the reverse scan is faster (see Figure 2a,b), and the numerical value of the specific capacitance is also higher in the dilute electrolyte compared to the neat IL.

The CDC-NbC curves appear relatively similar regardless of the neat or dilute electrolyte used (Figure 5c,d). In both electrolytes, the increase in cathodic current at critical potentials is lower compared to CDC-TiC and CDC-Mo_2_C. However, in the neat IL, there is an inexplicable peak (−1.0 V) during the reverse scan of the cathodic region (also observed in the −1.7 V curve), which is not present in the dilute IL electrolyte. Additionally, in both electrolytes, there is a lower cathodic current peak during the reverse scan, around −0.5 V, which is characteristic of all studied carbons. The anodic curves for CDC-NbC show differences compared to the neat and dilute ILs. In the first one, after +1.7 and +1.8 V, there is also a flat peak around +0.8 V, which could be attributed to the desorption of TFSI^−^ anions after passing higher potentials due to their specific adsorption in CDCs with multi-modal pores (i.e., CDC-NbC and CDC-Mo_2_C), though this is not confirmed. In the diluted electrolyte, with a lower ion concentration, such a distinct peak does not seem to form with either material.

In the case of CDC-Mo_2_C (Figure 5e,f), the cathodic curve in the neat IL resembles the shape of the CDC-TiC dependence on the CV curves obtained in the EMIm-TFSI/ACN electrolyte. Despite the differences in porosities, it appears that solvated cations in CDC-TiC and CDC-Mo_2_C exhibit similar electrosorption properties. The anodic curves of CDC-Mo_2_C are clearly different depending on the electrolyte. For instance, in the neat IL, a flat peak forms around +1.0 V during the anodic charging of the material after passing a wider potential window > −1.6 V. However, a similar, albeit very small, peak also forms for CDC-NbC after −1.8 V. However, the cathodic CV curves in CDC-Mo_2_C are very similar in both electrolytes, except for a slight difference in the sharper increase in current at −1.8 V during cathodic charging in the neat IL compared to the dilute IL. For CDC-Mo_2_C, the anodic CV curves are similar to the anodic curves of CDC-NbC in the diluted electrolyte. However, there is a significant difference after +1.8 V potential, similar to the cathodic potential, indicating that this material has a somewhat smaller electrochemical window in the ACN-containing electrolyte.

**Figure 6:**
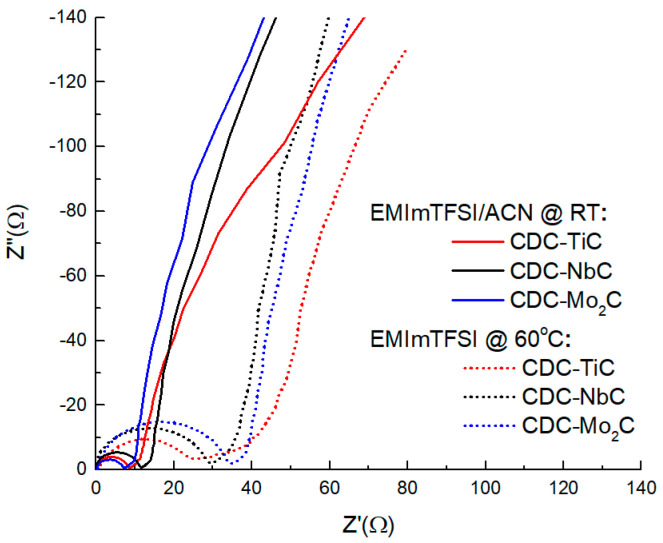
Nyquist plots of CDC materials in neat EMIm-TFSI at 60 °C and 1.9 M EMIm-TFSI/ACN at room temperature (RT).

The Nyquist plot in different electrolytes (Figure 6) shows a clear differentiation of the curves into two groups (in the neat IL electrolyte characterized by larger semicircles and in the diluted solvent with smaller semicircles). The larger radius of the semicircle is clearly due to the IL electrolyte having nearly 3× higher viscosity and about 2× lower electrical conductivity compared to the ACN electrolyte. In the neat IL, the plot also reveals material-related differences; for instance, the size of the semicircle (related to the charge transfer resistance) is clearly correlated with the material’s porosity and the size of the material’s particles, which, in the present case, both increase in the order CDC-TiC (Ø < 4 μm), CDC-NbC (Ø < 10 μm), and CDC-Mo_2_C (Ø < 20 μm). In addition to the semicircle, the most microporous material also has the largest area of so-called pore resistance, with an angle measuring less than 45°. This lower angle characterizes the simultaneous occurrence of mixed kinetic processes (partially impeded ion diffusion processes and adsorption processes). The other two materials exhibit a noticeable 45° rise. In both electrolytes, the vertical section of the CDC-TiC material does not reach a height of 90° due to steric hindrance to the electrosorption of electrolyte ions in the nanoporous material.

The *R*_s_ values calculated for the uncharged material at a frequency of 1 kHz reveal that the ultramicroporous CDC-TiC has very high electrical resistance (11.4 ohms) in the neat IL electrolyte. This is due to hindered diffusion as observed in the section of the “45-degree slope” (so-called pore resistance), on the Z′,Z″ curve in Figure 6, which also causes heat to be released during electrochemical cycling. However, in general, the capacitance of all three CDCs is similar, manifesting in the range of 3.1–5.6 ohms, and it does not matter if they are in solution or not.

**Figure 7:**
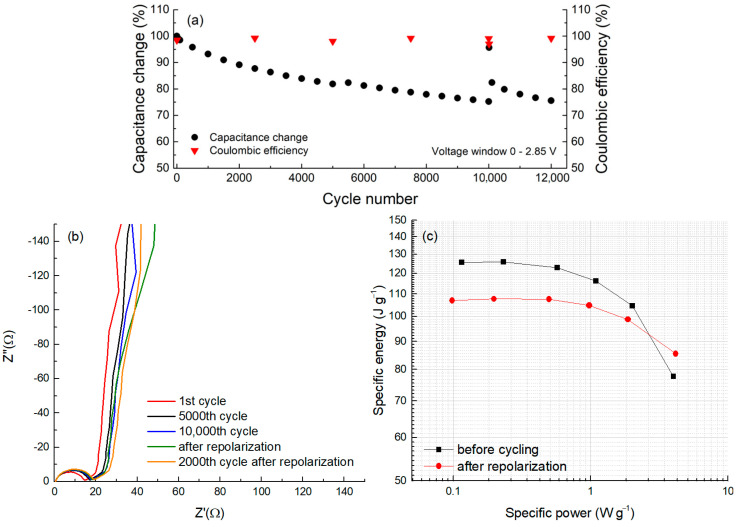
Capacitance and coulombic efficiency evolution (**a**), EIS spectra (**b**), and a Ragone plot (**c**) in cycle life test with a 2-electrode CDC-NbC cell at a voltage scan rate of 50 mV s^−1^ (**a**), including short-term repolarization after 10,000 cycles.

For cycle life testing, a symmetric two-electrode cell assembled from CDC-NbC electrode discs was subjected to cycling between 0 and 2.85 V using CV. The evolution of capacitance over the cycles is depicted in Figure 7a. After completing 10,000 cycles, we reversed the cell’s terminal polarities, followed by 10 cyclic voltammetry (CV) cycles from 0 to 2 V, referred to as ‘repolarization.’ Subsequently, we restored the original polarities, and cycling continued within the voltage range of 0 to 2.85 V. During the 10,000 cycles, the capacitance exhibited a reduction of approximately 22% from the initial value of 122 F g^−1^. This gradual reduction in capacitance over time is a hallmark of most electric double-layer (EDL) capacitors.

Immediately after repolarization, the capacitance recorded in the first cycle exhibited a remarkable surge, almost returning to the initial capacitance level (98%). However, this increase was transient, as subsequent cycling resulted in a rapid decline. This “overshoot” phenomenon can be attributed to the “cleaning” of the electrode surfaces from adsorbed compounds following the polarity reversal [44]. The freshly exposed surface momentarily regains its capacity for readsorbing electrolyte ions. Nevertheless, the redeposition of additives occurs quite swiftly, and the attained capacitance level proves to be temporally unstable.

In addition to the capacitance data obtained via the CV method, this test cell was subjected to electrochemical impedance spectroscopy after varying numbers of cycles (Figure 7b).

In Figure 7b, a Nyquist plot is presented, obtained before and after a certain number of full charge–discharge cycles. In this plot, the semicircles start from a single point, indicating a very stable system and a constant resistance at the metal/carbon electrode interface. Thus, changes in the carbon/aluminum resistance during the cycle life test can be ruled out. The semicircle slightly grows after around 5000 cycles, suggesting some alterations occurring on the carbon surface. However, the pore resistance (45° rise and its length) remains relatively stable, indicating that any potential changes over time are likely taking place on the external surface of the carbon particles. Furthermore, when observing the vertical “tail”, it becomes less vertical just after repolarization (Figure 6b, green curve) compared to the others, suggesting that this process might induce some parallel reaction in the system. Nevertheless, these processes (reactions) ceased to manifest after 2000 cycles of post-repolarization. It is possible that during cycling, surface oxides may attach to cations through anodic hydrogen-bonding-like interactions, hindering anion electrosorption. However, during the repolarization process, the breaking of these hydrogen bonds occurs, resulting in the observed changes. 

The values of the series resistance (*R*_s_) measured at 1 kHz are 12.8, 14.2, and 14.5 ohms after the 1st, 5000th, and 10,000th cycles, respectively. Immediately after repolarization, an *R*_s_ of 14.9 ohms was measured, with 15.2 ohms after the next 2000 cycles. Therefore, the overall change in cell resistance after completing 12,000 cycles is approximately 19%; this represents only one-fifth of the allowable change in the R_s_ value, as stipulated by conventional lifetime-testing agreements. In contrast, the decrease in capacitance over the same number of cycles is approximately 20%.

Figure 7c represents the dependence of energy density on the applied power density, also known as the Ragone plot, at the beginning of the cycle life test and immediately after repolarization. The data on the Ragone plot were derived from the dependencies on different cycling rates using the CV method. It was shown that the maximum usable energy of CDC-NbC in a symmetric two-electrode cell at low power is 125 J g^−1^, which decreases to nearly 108 J g^−1^ after 10,000 cycles (includingrepolarization) at an operating voltage of 2.85 V. The observed increase in power density after 10,000 cycles, particularly at high currents, might be associated with repolarization and the rapid surge in capacitance.

## 3. Materials and Methods

### 3.1. Synthesis and Characterization of CDC Materials

The CDC samples of variable pore size distribution were made by a high-temperature chlorination of TiC (H.C. Starck, Ø < 4 µm), NbC (Alfa Aesar, Ø < 10 µm), and Mo_2_C (Reahim, Ø < 20 µm) at 500 °C, 1000 °C, and 800 °C, respectively. Carbide powder, placed in the horizontal quartz tube, was reacted with chlorine (Linde Gas, 2.8, 1.5 L min^−1^) flow at a fixed temperature. During heating and cooling, the reactor was purged with argon (Linde Gas, 4.0, 2 L min^−1^) flow. After chlorination, the CDC product was additionally treated with hydrogen (Linde Gas, 4.0, 1 L min^−1^) at 800 °C to deeply dechlorinate the sample. The detailed synthesis conditions of CDC materials are described elsewhere [2].

Textural properties of CDC materials were determined by low-temperature N_2_ adsorption at 77 K and CO_2_ adsorption at 273 K using a NOVAtouch LX2 (Quantachrome Instruments, Boyton Beach, FL, USA). The BET surface area (*S*_BET_) was evaluated based on the BET theory in the *P*/*P*_0_ interval of 0.02–0.2, and the total pore volume (*V*_tot_) was calculated at *P*/*P*_0_ of 0.97. The specific surface area (*S*_dft_), pore size distribution (PSD), and volume of micropores (*V*_µ_) were calculated from N_2_ adsorption isotherms using a quenched solid density functional theory (QSDFT) equilibrium model for slit-type pores. The PSD from CO_2_ adsorption was determined using a non-local density functional theory (NLDFT) model for slit-type pores. In all calculations, the software TouchWin ver. 1.1 was used.

### 3.2. Electrochemical Characterization 

Electrochemical characterization of carbon materials was performed in three-electrode test cells. Working electrodes with a visible surface area of 0.38 cm^2^ were made by pressing a slurry of 90% CDC and 10% PTFE (Aldrich, Taufkirchen, Germany, 60% dispersion in water) into a carbon sheet. The thickness of the working electrodes was ~100 µm. The reference and counter electrodes were made from high-surface-area carbon sheets, which, for better electrical contact, were coated on one side with a 2 µm layer of pure Al using PVD. The counter and working electrodes were separated with two layers of SWP glass mat-type separator (Anpei, Tainan, Taiwan, thickness ~1 mm). All electrodes and separators were preliminary dried in vacuum (1 mbar) at 110 °C for a week. After assembling, the cell was vacuumed overnight at 110 °C and filled with the electrolyte 1-ethyl-3-methylimidazolium bis(trifluoromethylsulfonyl)imide (EMIm-TFSI, Sigma-Aldrich, Taufkirchen, Germany, ≥98%) and a 1.9 M solution of EMIm-TFSI in acetonitrile (Sigma-Aldrich, anhydrous 99.5%). The density of the electrolyte solution was 1.18 g cm^−3^. The neat ionic liquid was used as is, and the IL/ACN mixture was kept on molecular sieves (3 Å) for one week before use. Measurements with neat ionic liquid were performed at 60 °C and with EMIm-TFSI/ACN at room temperature; in both cases, the test cell was soaked for 24 h before measurement.

The electrochemical characteristics of carbon materials were measured using cyclic voltammetry (CV), galvanostatic (CC), and electrochemical impedance spectroscopy (EIS) methods. The potentiostat–galvanostat 1286 with FRA 1255B (Solartron, Hampshire, UK) was used for all electrochemical measurements. The integral capacitance (*C_i_*) was calculated from CC experiments according to the equation *C_i_* = *I*Δ*t*/Δ*U*, where *I* is the discharge current and Δ*t* is the discharge time in the potential range of Δ*U*. The specific capacitance values of CDC ((F g^−1^), (F cm^−3^)) were calculated as *C*_CC_ *= C*_i_/*m* and *C*_CC_ = *C*_i_/*V*, where *m* is the weight of the CDC in a working electrode and *V* is the volume of the working electrode.

The cycling stability of CDC-NbC was evaluated in a two-electrode cell assembled from two electrodes of 7 mm in diameter separated with two layers of cellulose separator TF44-25 (Nippon Kodoshi Corp., Nankoku-City, Kochi, Japan). The CV applying a voltage scan rate of 50 mV s^−1^ was used to evaluate the cycling life in the voltage ranges of 0–2.85 V. Prior to the test, the cell was preconditioned with CV cycling (*v* = 50 mV s^−1^), expanding the potential window from 0–1.2 V to 0–2.85 V with an interval of 0.2 V. The capacitance was calculated from CV plots as differential capacitance *C_D_* = *j*/*ν* = *j*(d*E*/d*t*)*,* where *j* is the current at a fixed cell voltage (1.5 V) and *ν* is the voltage scan rate. The CV data were used to construct the experimental Ragone plots (energy density vs. power density). The coulombic efficiency was calculated as *ƞ* = *Q* (discharge)/*Q* (charge), where *Q* is the charge. The changes in resistance were measured by EIS at 1 kHz using the ZView ver. 3.5i software (Scribner Associates Inc., Southern Pines, NC, USA).

## 4. Conclusions

In this study, three carbide-derived carbon materials of TiC, NbC, and Mo_2_C origin, characterized by uni-, bi-, and tri-modal pore sizes, respectively, were investigated for energy storage in both neat EMIm-TFSI and 1.9 M ACN-diluted EMIm-TFSI electrolytes. To elucidate the relationships between porosity and the electrochemical properties of carbon materials, cyclic voltammetry, galvanostatic cycling, and electrochemical impedance spectroscopy measurements were performed using three-electrode test cells. It was demonstrated that micromesoporous CDC-Mo_2_C, with a large fraction of 3–4 nm sized pores, has good access to micropores and, therefore, excellent gravimetric capacitance in both neat and dilute ILs. However, the low packing density of CDC-Mo_2_C results in the low energy density of the corresponding storage device, which makes it impractical for large-scale production. Ultramicroporous CDC-TiC exhibits a narrow pore size distribution at approximately 0.8 nm. This carbon material is distinguished by its high packing density of 0.85 g cm^−3^ and demonstrates a notable volumetric capacitance of 93.6 and 75.8 F cm^−3^ for the negatively and positively charged electrodes in the dilute IL, respectively. Regrettably, the significant pore resistance arising from the absence of transport pores results in elevated overall impedance for the associated energy storage device. This elevated impedance is undesirable in numerous applications, particularly in sectors such as transportation. The bi-modal pore-sized microporous CDC-NbC has a main fraction of micropores with the same size as CDC-TiC. And although it has a slightly lower capacitance of 85.0 and 73.7 F cm^−3^ for the negatively and positively charged electrodes in the dilute IL, respectively, the lower pore resistance due to the additional fraction of 1–2 nm micropores makes it more suitable for real-world applications. A symmetric two-electrode capacitor incorporating microporous CDC-NbC electrodes revealed an acceptable cycle life. After 10,000 cycles, the cell retained ~75% of its original capacitance, while the ESR increased only by 13% at a coulombic efficiency of 99%.

## Figures and Tables

**Table 1:** Specific surface area (*S*_BET_ and *S*_dft_) including micro- (*S*_dft,µ_) and mesopore (*S*_dft,meso_) ratios, total pore volume (*V*_t_), and volume of different pore size fractions (*V*_< x nm_) calculated from N_2_ adsorption isotherms.

ID	*S*_BET_ (m^2^ g^−1^)	*S*_dft_ (m^2^ g^−1^)	*S*_dft,µ_ (m^2^ g^−1^)	*S*_dft,meso_ (m^2^ g^−1^)	*V*_tot_ (cm^3^ g^−1^)	*V*_1nm_ (cm^3^ g^−1^)	*V*_1.5nm_ (cm^3^ g^−1^)	*V*_2nm_ (cm^3^ g^−1^)
CDC-TiC	1257	1318	1306	12	0.61	0.47	0.52	0.53
CDC-NbC	2247	1893	1765	128	1.17	0.40	0.68	0.9
CDC-Mo_2_C	2204	1951	1356	595	1.67	0.32	0.48	0.69

**Table 2:** Specific capacitance of CDCs at positively and negatively charged electrode potentials by CC (0.5 mA cm^−2^) in EMIm-TFSI electrolyte.

Carbon Sample	$C_{cc}^{-}$ (F g^−1^)	$C_{cc}^{+}$ (F g^−1^)	$C_{cc}^{-}$ (F cm^−3^)	$C_{cc}^{+}$ (F cm^−3^)
CDC-TiC	127.3	113.3	99.9	88.9
CDC-NbC	145.1	110.0	81.6	61.8
CDC-Mo_2_C	140.1	146.0	63.2	65.9

**Table 3:** Specific capacitance of CDCs at positively and negatively charged electrode potentials by CC (0.5 mA cm^−2^) in EMIm-TFSI/ACN electrolyte.

Carbon Sample	$C_{cc}^{-}$ (F g^−1^)	$C_{cc}^{+}$ (F g^−1^)	$C_{cc}^{-}$ (F cm^−3^)	$C_{cc}^{+}$ (F cm^−3^)
CDC-TiC	137.6	113.0	93.6	75.8
CDC-NbC	138.3	119.8	85.0	73.7
CDC-Mo_2_C	140.0	127.9	64.8	59.2

## Data Availability

All data relevant to this publication are included.
